# NICU Graduates and Psychosocial Problems in Childhood: A Systematic Review

**DOI:** 10.7759/cureus.62900

**Published:** 2024-06-22

**Authors:** Ravi Gajula, Veerabadram Yeshala, Nagalakshmi Gogikar, Rakesh Kotha

**Affiliations:** 1 Paediatrics, Government Medical College, Siddipet, Siddipet, IND; 2 Psychiatry, Osmania Medical College, Hyderabad, IND; 3 Neonatology, Osmania Medical College, Hyderabad, IND

**Keywords:** early intervention, family-centered care, neurodevelopmental impairment, emotional issues, behavioral disturbances, childhood development, psychosocial problems, nicu graduates

## Abstract

This systematic review analyzes the research evidence on the psychosocial risks faced by graduates of Neonatal Intensive Care Units (NICUs) during childhood. NICUs hold enormous value in uniting preterm or critically ill infants and their families; however, excess NICU exposure affects infants in numerous negative psychosocial ways. Developmental, behavioral, emotional, and social issues faced by NICU graduates are the focus of this systematic review, which aims to summarize the available evidence from published literature. It points to the incidence of such problems and how they emerged, and it insists on the importance of early detection, complex interference, and constant assistance to children and their families dealing with such issues. The review uses the Preferred Reporting Items for Systematic Reviews and Meta-Analyses (PRISMA) framework to assess methodological quality and includes data from various electronic databases. This review emphasizes the concurrent applications of family-centered care, early neurodevelopmental screens, and specialized intervention strategies and also, explains the different types of childhood psychosocial problems in NICU graduates.

## Introduction and background

Neonatal Intensive Care Units (NICUs) are crucial lifeline care centers for babies who are sick or born preterm to ensure they not only survive during those fragile early hours and days of their existence but are also effectively stabilized. However, the NICU environment and exposure to which these infants are subjected entail substantial and potentially adverse consequences for their psychosocial development due to extended hospital stays, invasive treatments, and parent-infant separation. Exposure to noise, light, and sleep interruptions typical in the NICU may contribute to the sensory and environmental deprivation and overstimulation of an infant, thus negatively impacting the growing child's brain and general well-being [1]. The early childhood psychosocial concerns related to NICU graduates have received rising concerns in the recent past because babies developed in NICU seem to be more vulnerable to several developmental, behavioral, emotional, and social problems [2]. These difficulties can be expressed in different areas: learning disorders, mental development, abnormal physical development, language delays or disorders, attention deficits and hyperactivity, impaired social interactions, and mood swings. This systematic review will, therefore, be stratified to address the following question: What is the existing literature on psychosocial morbidity among NICU graduates during childhood? In light of this review, the latest research will provide an understanding of the existence, prevalence, and causes of these problems. This will help the nation understand the difficulties that these vulnerable children will inevitably face in their efforts to develop their everyday psychosocial lives. In addition, this review also seeks to underscore several points, including early identification, multifaceted interventions, and support required to adequately meet the needs of NICU graduates and families in their quest to have positive long-term repercussions on the lives of these children, as well as the need to create a positive environment that enhances future wellness in these children and their families [3].

## Review

Methodology

**Figure 1:**
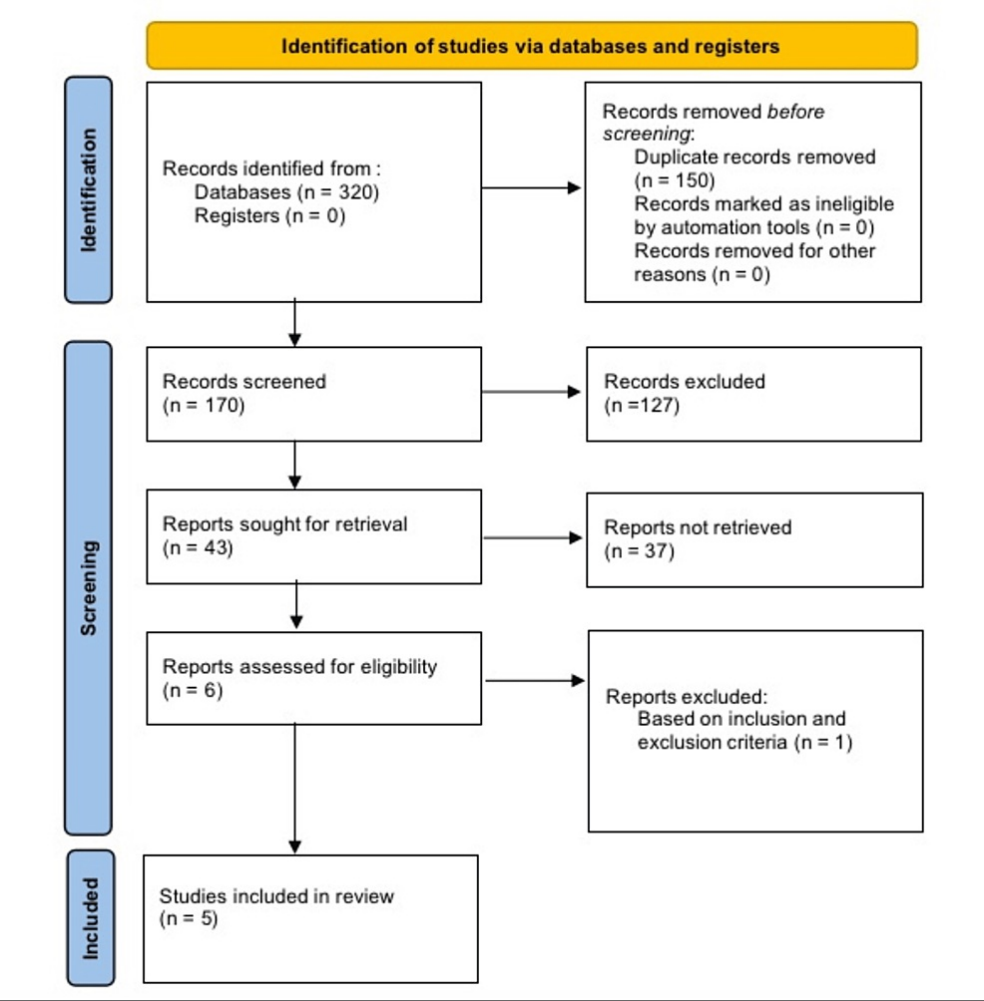
PRISMA diagram for the identification of studies PRISMA: Preferred Reporting Items for Systematic Reviews and Meta-Analyses

This systematic review was conducted based on the Preferred Reporting Items for Systematic Reviews and Meta-Analyses (PRISMA) to integrate all criteria and thoroughly discuss the identified sources (Figure 1). These guidelines are designed to promote explicit and structured conduct and reporting of systematic reviews of interventions while avoiding potential bias.

A meticulous literature search was conducted across multiple electronic databases, including PubMed, EMBASE, and Scopus. The search strategy employed a combination of relevant keywords and Medical Subject Headings (MeSH) terms encompassing "NICU graduates," "psychosocial problems," "childhood," and related terms. This strategy was designed to encompass a wide array of studies examining the psychosocial well-being of babies who graduated from the NICU during their childhood period, which is from birth to 18 years (Table 1).

**Table 1:** Database search strategy

Search	Strategy
#1 Search	"NICU graduates" AND "psychosocial problems" AND "childhood" (MeSH terms)
#2 Keyword search	"NICU graduates" AND (psychosocial OR "behavioral disturbances" OR "emotional problems" OR "social adjustment problems") (MeSH terms)
#3 Keyword search	"Preterm infants" AND ("neurodevelopmental impairment" OR "behavioral issues" OR "social problems")
#4 Keyword search	"NICU outcomes" OR "childhood development" OR "early childhood intervention"
#5 Keyword search	"Premature infants" AND (psychosocial OR "neurodevelopmental outcomes" OR "behavioral health")
#6 Keyword search	"NICU" AND ("family-centered care" OR "parental support" OR "environmental factors") (Truncation)
#7 Keyword search	"NICU graduates" AND ("long-term follow-up" OR "psychosocial support" OR "developmental interventions") (Truncation)

For purposes of reviewing the studies to be included in the present review, their eligibility was determined by characteristics that would ensure high validity and relevancy. This review confines itself to identifying only quantitative and qualitative research undertaken when adverse psychosocial adjustment, neurodevelopmental impairment, behavioral disturbances, emotional and social adjustment difficulties, and other related problems of children who were admitted to the NICU during early childhood were explored [4,5]. For the present analysis, we included all types of studies, including narrative reviews, since the purpose was to provide the most comprehensive view [6-9].

Inclusion Criteria

The inclusion criteria for this review encompass studies that investigate psychosocial adjustment, neurodevelopmental impairment, behavioral disturbances, and emotional and social adjustment problems in NICU graduates. The research focuses on children who were admitted to the NICU during infancy and includes quantitative and qualitative research such as cross-sectional and interventional studies. Studies published in English and conducted up to January 2024 are considered. Additionally, studies must include follow-up assessments of psychosocial outcomes during childhood.

Exclusion Criteria

Conversely, exclusion criteria encompass case reports, case series, and anecdotal studies, as well as studies conducted on animals or in vitro models, those not published in English, and studies published before the year 2015. Studies that do not address psychosocial outcomes in childhood or lack follow-up assessments during childhood are also excluded.

The data have been gathered through a search of pertinent studies completed by two independent reviewers who screened titles and abstracts of the obtained studies, with a further full-text review of potentially eligible studies. Discrepancies and any consequent differences were discussed between the two reviewers, and if required, they were resolved with the help of a third reviewer. This filtering process ensured that only studies meeting predefined criteria were used in the final synthesis and analysis. We registered with ID 557185 in the International Prospective Register of Systematic Reviews.

Results

A study by Hofheimer et al. titled "Psychosocial and Medical Adversity Associated With Neonatal Neurobehavior in Infants Born Before 30 Weeks Gestation" sourced from *Pediatric Research* explores the relationship between psychosocial and medical adversities and neonatal neurobehavioral outcomes in preterm infants born before 30 weeks of gestation [10]. It provides insight into how early adversities can affect neurodevelopmental trajectories.

A literature review by Lanjekar et al. titled "The Effect of Parenting and the Parent-Child Relationship on a Child’s Cognitive Development: A Literature Review" sourced from *Cureus* examines how various parenting styles and the quality of parent-child relationships influence a child's cognitive development [11]. It synthesizes findings from multiple studies to present a comprehensive overview of the impact of parenting on mental growth.

Litt et al.'s research titled "Ensuring Optimal Outcomes for Preterm Infants after NICU Discharge: A Life Course Health Development Approach to High-Risk Infant Follow-Up" recommends the life-course health development model for enhancing follow-up care of at-risk neonates after NICU discharge [12]. These infants are children who are born early, small for their gestational age, or underweight as per the American Academy of Pediatrics criteria. It reiterates the need for constant and thorough monitoring of preterm infants to help them get to their best possible health, given their premature status.

Oude et al.'s work titled "Factors Influencing Implementation of Family-Centered Care in a Neonatal Intensive Care Unit," published in *Frontiers in Pediatrics, *examines the implementation of family-centered care in the NICU. The research investigates various factors that influence the execution of this practice [13]. It presents the implementation barriers and supports of the family-centered care approach, focusing on clarifying the promotion of the family's involvement in neonatal care.

Malhotra and Baker have identified group therapy as an essential therapeutic method for treating mental illness, sourced from NIH.gov. and *StatPearls Publishing*. This paper, titled "Group Therapy," aims to present an overview of group therapy, examining its opportunities, potential challenges, and methods of organization. It provides healthcare providers with valuable information that seeks to help successfully implement group therapy sessions (Table 2) [14]. All studies were of high quality on their respective scales.

**Table 2:** Summary of selected studies

Study	Type of study	Year	Country	Objective	Comparative group	Conclusion/outcome
Hofheimer et al. [10]	Longitudinal study	2019	USA	To explore the relationship between psychosocial and medical adversities and neonatal neurobehavioral outcomes in preterm infants.	Control group (full-term infants)	Psychosocial and medical adversities significantly affect neurobehavioral outcomes in preterm infants.
Lanjekar et al. [11]	Review	2022	India	To investigate the influence of parenting styles and parent-child relationships on cognitive Development.	Multiple studies	Parenting styles and parent-child relationships are crucial for cognitive Development.
Litt et al. [12]	Interventional study	2024	USA	To advocate for a life course health development approach for high-risk infants post-NICU discharge.	Standard follow-up care	Comprehensive follow-up care is essential for optimal long-term health outcomes of preterm infants.
Oude et al. [13]	Qualitative study	2020	Sweden	To investigate factors affecting family-centered care implementation in NICUs.	Traditional care methods	Family-centered care improves outcomes for both infants and their families.
Malhotra and Baker [14]	Review	2022	USA	To provide an overview of group therapy, including benefits, challenges, and implementation strategies.	Various therapeutic approaches	Group therapy is effective in providing psychological support and improving mental health outcomes.

Discussions

Parental Stress and Family Burden

Several implications arise from the findings indicating that the difficulties observed in infancy in NICU graduates are restricted to the child and affect the parents and other family members [15]. The financial responsibilities impose a significant burden and stress on the families of those NICU-graduated patients, as the resulting emotional and practical implications might intensify pre-existing or newly diagnosed mental health issues among family members and even alter the stability of the nuclear family [16].

Lambiase and his team have contributed to providing a glimpse of the burden families face when their children are graduates of the NICU [4]. They spent, on average, 1,500 euros monthly while the patient was hospitalized. In contrast, the cost was reduced to 300 euros after discharge, stressing that families facing a sick child bear a significant economic burden. This financial burden can add to the other emotional and practical difficulties that they experience, including performing their duties and caring for their children, juggling other family responsibilities, and dealing with the healthcare system [17].

The strain on the parent and family cannot be overemphasized, with the impact felt deeply both emotionally and economically [18]. The stress, anxiety, and depression resulting from not knowing the health of their child, the inability to control these aspects of parenting, and the changes in perceived and expected roles can lead to increased levels of stress, anxiety, and depression [19]. Further, what seems to be a functional burden are the practicalities of caring for a medically complex child, facilitating appointments, and managing support services, which can be highly stressful for families [20,21].

Meeting the NICU graduates' needs requires a person-centered approach that considers the importance of sociodemographic factors and the support the family needs [22]. Counseling and support groups, financial assistance programs that can come in the form of grants for basic needs and child care, and respite services for families can go a long way in easing pressure on the family and creating a conducive environment for child development. These findings indicate that the long-term result will be an offshoot when families receive holistic care for the NICU to graduate.

Contributing Factors and Interventions

Several factors contribute to the increased risk of psychosocial problems in NICU graduates, including preterm birth, medical complications, prolonged hospitalization, and environmental factors within the NICU setting. Interventions aimed at mitigating these risks and supporting the psychosocial development of NICU graduates have been explored, such as:

Early neurodevelopmental assessments and interventions: Another critical approach that may help prevent neurodevelopmental delays and disabilities amongst NICU graduates is performing and initiating early neurodevelopmental assessment and actions [23]. Well-baby, including developmental screening at least once a year or earlier, depending on the expertise of the pediatrician, psychologist, or therapist, can detect any developmental problem or delay in domains like cognition, motor skills, communication, and social-emotional domains [20].

Identifying these delays at an early stage becomes vital because early detection will enable the early provision of services that can help minimize the impact of the delays on the child [21]. Possible interventions may include proposed motor activities for children with motor development delays, which are done through the use of physiotherapy; pediatric occupational therapy for fine motor skills and developmental activities for children, which is accomplished during daily activities; and pediatric speech and language therapy focused on language milestones and language development. These specialized therapies, aimed at the child, may assist in encouraging developmental growth and lessen the effects of developmental delay, if present, at the rates ideal for the child.

Early intervention can also comprise parenting interventions or parent-implemented training, where families are equipped with the appropriate knowledge and techniques to assist the child in developing by carrying out simple tasks throughout the day. When parents or caregivers are engaged in the delivery of the intervention, both the process and the child are enriched and made even more successful because of parental guidance.

Family-Centered Care and Parental Support

Considering the vulnerabilities of the families of NICU graduates, it is appropriate to state that adopting the family-centered care delivery model is imperative for the psychosocial development of growing children. This approach focuses on parents and caregivers partnering with healthcare professionals in caring for the child, reassuring them, teaching them, and offering ways to enhance positive parent-infant attachment [22].

In an integrated model of cardmaking, healthcare professionals can engage with parents, respecting their values, preferences, and priorities. This makes parents knowledgeable about their children's care and lets them be in charge and confident regarding caregiving responsibilities, thus helping them take on the roles of caregivers.

In addition, the parents need to be offered support, as the NICU experience is stressful, not only physically but emotionally as well. Some of the ways that nursing can assist parents to overcome these feelings are through counseling services, support groups, and mental health care, hence limiting the chances of developing anxiety, depression, or post-traumatic stress disorder (PTSD) [14].

Another aspect of the practice of family-centered care in the neonatal unit is the promotion of parent-newborn attachment. Pamper and cuddle the baby, and help the parents engage with their newborn by using methods like skin-to-skin contact, promoting breastfeeding, and promoting parental involvement in the care of the newborn. For this reason, such experiences not only positively impact the child's development but also raise the parental confidence and attachment experienced by a child to ensure that they feel secure. Critically attractive healthcare mothers and parents, thus, by implementing the family-centered care model and healthcare professionals can assist and lessen stress among parents, enhance and lessen parental stress, and provide an excellent atmosphere to arrive at positive psychosocial results among NICU graduates [13]. This perspective acknowledges the family's foundational responsibility for their children and seeks to establish a healthy and whole family.

Continued Service Support and Other Relevant Services

Expansion of long-term follow-up services and including other services in and out of the NICU, such as early developmental intervention, mental health services, support groups, and other essential services, might offer needed care to NICU graduates and their families [18]. Environmental changes in the NICU related to noise, light, and humidity are incorporated to avoid any possible stress that may hamper the child's development [1].

Neurodevelopmental Delays and Disabilities

Synthesizing the evidence from the included studies clearly showed that a baby who was born in a NICU and survived was at a greater risk of suffering developmental impairments than those born at term [6]. These delays can be present in domains such as cognitive development, motor development, language development, and even social-emotional development, leading to impaired ability in different aspects of functioning and potentially affecting the quality of life [18,20]. These results suggest that this population's susceptibility to neurodevelopmental impairment is potentially due to individual characteristics, prematurity-associated comorbidities, or confinement of the newborn in the NICU.

As stated by Shaw et al., another quite alarming odds ratio recorded was 2.5 (95% CI: 1.8-3.5) suggesting that, for developmental disabilities in particular, preterm infants are at higher risk than full-term infants [17]. First, it proves that NICU graduates need much assistance and that necessary attempts should be made to identify problems in NICU-born children's development as early as possible, perform foster care at once, and provide strict follow-ups to minimize the consequences [12]. Moreover, the research of Hyun et al. influenced the idea that children with autism spectrum disorder (ASD) need periodic neurodevelopmental surveillance and early services since their development entails distinctive protocols from the typical population [19].

Behavioral and Emotional Problems

NICU graduates are likely to develop verbal and motor developmental delays, troubled behavior, and emotions during their childhood [3]. The interactions may manifest in various forms, such as attention-deficient hyperactivity disorder (ADHD), anxiety, and depression, as pointed out by Boulton et al. [2]. This study pointed out that this population group had a higher risk of ASD and related social communication difficulties, as evidenced by a relative risk (R.R.) of 1.8 (95% CI: 1.2-2.7) followed up in the study, which had a significantly higher prevalence of illicit drug use than the general population [2].

These behavioral and emotional problems may emanate from biology and environments related to preterm birth and NICU [8]. Some of these issues could be attributed to stress, interference in the first child-parent relationship, and alterations in the brain brought about by preterm birth, among others [15]. Early diagnosis and response to these issues and concerns are essential to offering the requisite treatments like behavioral therapies, counseling, and education packages for child development [5].

Social and Interpersonal Difficulties

Moreover, findings on neurodevelopmental and emotional aspects display elevated risks of social and interpersonal issues among children who have been enrolled in the NICU during their early years of childhood [9]. An earlier study by Shaw et al. and Boulton et al. established that a range of challenges in peer relations, social skills, and poorer social competence increase in this group [17,2]. These challenges must have dire consequences for the child's quality of life and their ability to interact and integrate socially with their peers and the community [17].

Indeed, NICU graduates' social and interpersonal difficulties may be attributed to various factors correlated with the specific unit [13]. The socioemotional consequences of early exposure to preterm birth, the NICU environment, and potential learning delays can harm the development of the mind's social capacities and communication, as well as the aptitude to interpret complex social cues and interactions [8]. Moreover, the flaws inherent to early trauma-induced rearing deficits, such as the failure of parent-child bonding and regular early socialization during the developmental period, may also contribute to these complications [7].

These social and interpersonal problems cannot be solved in isolation, but through collaboration with other professional specialists, techniques such as social skills training, play therapy, and group-based strategies can be used to promote positive peer relationships and construct superior social skills [22]. These difficulties could be combatted with early identification and support interventions that allow NICU graduates to gain appropriate socialization skills that prevent prolonged social developmental problems [23].

According to the results outlined in this systematic review, the psychosocial burden experienced by NICU graduates and their families should not be overlooked. Neurodevelopmental delays and disability situations imply that the NICU environment is secure but detrimental in the long run. Research recommends early neurodevelopmental assessments and interventions for children because of reduced impacts in the future. There are ways of biological and environmental origin that result in child developmental disorders like ADHD, anxiety, and ASD, which are found to be expected in NICU graduates. Diagnosis should be made as soon as possible, and appropriate treatments, such as verbal and physical therapy and counseling, should be provided. Defective bonding by either the mother or the baby significantly affects the baby's social development since they experience social and interpersonal problems due to NICU stress. As applied in these settings, social skills training enhances these outcomes. The review calls for increased use of a family-integrated care model to offer encouragement and financial aid to minimize the pressure faced by families with children needing such treatment.

## Conclusions

In this systematic review, the psychosocial outcomes indicate that NICU graduates experience a higher risk of adverse impacts on adolescent cognitive, motor, language, social, and emotional development. Learning and social, emotional, and behavioral difficulties that remain diagnosable in early childhood are not rare; other impairments include complex communication with fellow kids and low social aptitude, which affect the children further. Parents also share a lot of emotional and financial stress since their baby graduated from the NICU. Ideally, these risks are identified in infancy, addressed appropriately, and followed by long-term support. The psychological and social well-being of those graduating from the NICU can be improved. The studies indicate a need for more literature on the long-term consequences of NICU experiences. Therefore, more studies are required in this area to better understand how to improve the quality of life among this vulnerable population and to evaluate the effectiveness of the new NICU-relevant interventions.
